# Endoscopic submucosal dissection for a small high grade intraepithelial neoplasia in the hypopharynx detected incidentally by artificial intelligence

**DOI:** 10.1055/a-2208-3412

**Published:** 2023-12-12

**Authors:** Ruide Liu, Xianglei Yuan, Shuang Liu, Bing Hu

**Affiliations:** 1 34753 Department of Gastroenterology and Hepatology, West China Hospital of Sichuan University, Chengdu, China

Hypopharyngeal carcinoma, a malignant tumor with a poor prognosis, is usually diagnosed at an advanced stage owing to underdiagnosis. Limited knowledge of hypopharyngeal lesions and rapid access to the esophagus during endoscopy might contribute to this delayed diagnosis 1 . Previous studies have shown the potential of artificial intelligence (AI)-based systems for high sensitivity detection of early pharyngeal cancer 2 . Here, we present a case in which a hypopharyngeal precancerous lesion was incidentally detected using AI and successfully treated by endoscopic submucosal dissection (ESD).

A 58-year-old man was referred to our hospital for endoscopic treatment of an esophageal mucosal lesion. He had experienced hoarseness and throat discomfort for 2 years. Enhanced computed tomography of the head and neck showed no obvious thickening or abnormal enhancement in the pharynx, nor any enlarged cervical lymph nodes ( Fig. 1 **a** ). During endoscopic treatment for the esophageal lesion, with the assistance of an AI system, a suspected hypopharyngeal precancerous lesion, approximately 0.7 cm in size, was detected in the corniculate tubercle of the hypopharynx ( Fig. 1 **b,c** , Video 1 ). Given the previous success of ESD in treating early hypopharyngeal cancer, the patient agreed to undergo the procedure, which was successfully performed 3 4 ( Fig. 1 **d** ). Histopathology revealed a high grade squamous intraepithelial neoplasia measuring 0.7×0.3 cm ( Fig. 2 ).

**Fig. 1:**
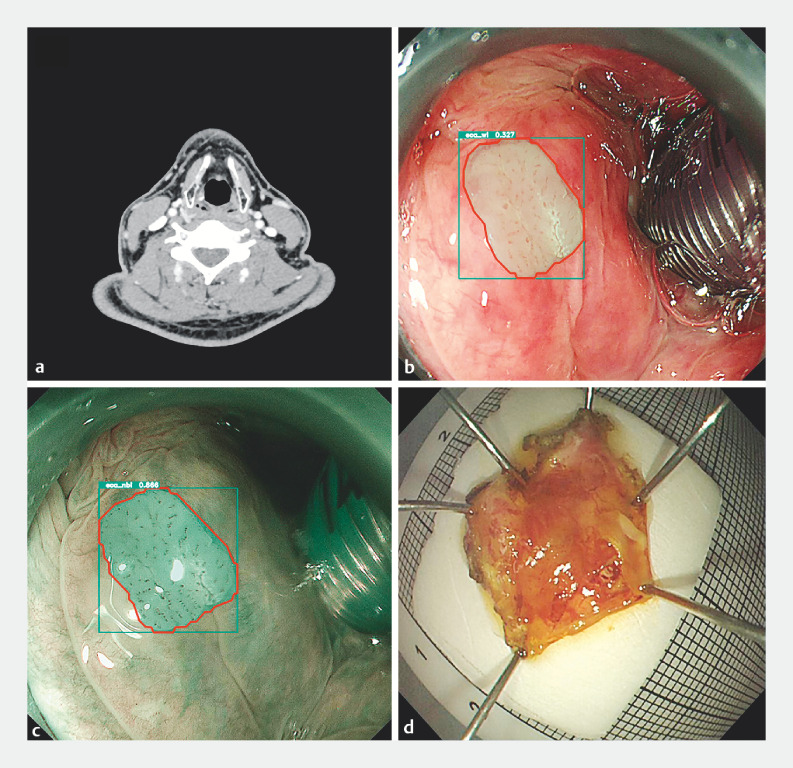
Preoperative images and the postoperative specimen. **a** Computed tomography showed that there was no obvious thickening or abnormal enhancement in the pharynx, nor any enlarged cervical lymph nodes. **b** Artificial intelligence system incidentally detected a small mucosal lesion of approximately 0.7 cm under white-light imaging. **c** The same lesion under narrow-band imaging. **d** The resected specimen measured 1.0 × 1.2  cm.

Endoscopic submucosal dissection for a small, high grade intraepithelial neoplasia in the hypopharynx detected incidentally by artificial intelligence.Video 1

**Fig. 2:**
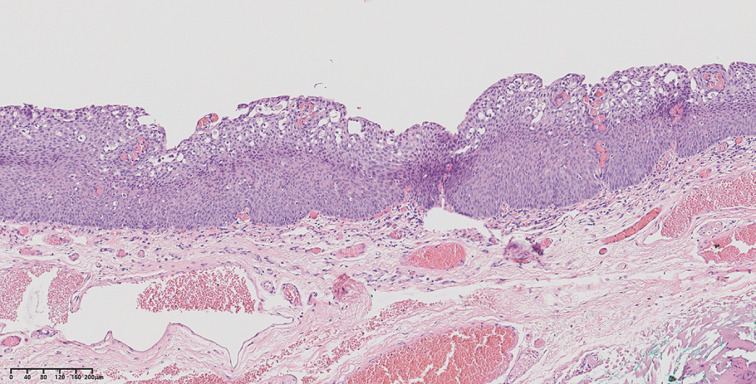
Histopathology of the specimen showed a high grade squamous intraepithelial neoplasia (hematoxylin and eosin ×200).

Subsequent follow-up endoscopy at 1 year showed no residual lesion or recurrence.

The potential for synchronous or metachronous head–neck cancer in patients with esophageal squamous cell carcinoma (ESCC) underscores the significance of careful examination of the pharynx during endoscopy in such patients 5 . Previous AI systems have primarily focused on detecting early hypopharyngeal cancer, leaving uncertainty about their capacity to identify hypopharyngeal precancerous lesions. While our team’s AI system was originally developed for ESCC and precancerous lesions, it has demonstrated efficacy in detecting similar squamous epithelial precancerous lesions in the hypopharynx. Nevertheless, further clinical studies are essential to validate its effectiveness.

Endoscopy_UCTN_Code_CCL_1AB_2AB

## Notes

Endoscopy E-Videos https://eref.thieme.de/e-videos
*E-Videos* is an open access online section of the journal *Endoscopy* , reporting on interesting cases and new techniques in gastroenterological endoscopy. All papers include a high-quality video and are published with a Creative Commons CC-BY license. Endoscopy E-Videos qualify for HINARI discounts and waivers and eligibility is automatically checked during the submission process. We grant 100% waivers to articles whose corresponding authors are based in Group A countries and 50% waivers to those who are based in Group B countries as classified by Research4Life (see: https://www.research4life.org/access/eligibility/ ). This section has its own submission website at https://mc.manuscriptcentral.com/e-videos .
